# Intraprocedural left ventricular free wall rupture diagnosed by left ventriculogram in a patient with infero-posterior myocardial infarction and severe aortic stenosis

**DOI:** 10.1186/s12872-016-0302-7

**Published:** 2016-06-06

**Authors:** Takao Konishi, Naohiro Funayama, Tadashi Yamamoto, Hiroshi Nishihara, Daisuke Hotta, Kenjiro Kikuchi, Hideo Yokoyama, Katsumi Ohori

**Affiliations:** Department of Cardiology, Hokkaido Cardiovascular Hospital, 1-30, West 13, South 27, Chuou-ku, Sapporo, 064-8622 Japan; Department of Translational Pathology, Hokkaido University School of Medicine, Sapporo, Japan; Department of Cardiovascular Surgery, Hokkaido Cardiovascular Hospital, Sapporo, Japan

**Keywords:** Left ventricular wall rupture, Acute myocardial infarction, Aortic stenosis, Angioplasty, Intra-aortic balloon pump, Catecholamine

## Abstract

**Background:**

Left ventricular wall rupture remains a major lethal complication of acute myocardial infarction and hypertension is a well-known predisposing factor of cardiac rupture after myocardial infarction.

**Case presentation:**

An 87-year-old man was admitted to our hospital, diagnosed as acute myocardial infarction (AMI). The echocardiogram showed 0.67-cm^2^ aortic valve, consistent with severe aortic stenosis (AS). A coronary angiography showed a chronic occlusion of the proximal left circumflex artery and a 99 % stenosis and thrombus in the mid right coronary artery. During percutaneous angioplasty of the latter, transient hypotension and bradycardia developed at the time of balloon inflation, and low doses of noradrenaline and etilefrine were intravenously administered as needed. The patient suddenly lost consciousness and developed electro-mechanical dissociation. Cardio-pulmonary resuscitation followed by insertion of an intra-aortic balloon pump (IABP) and percutaneous cardiopulmonary support were initiated. The echocardiogram revealed moderate pericardial effusion, though the site of free wall rupture was not distinctly visible. A left ventriculogram clearly showed an infero-posterior apical wall rupture. Surgical treatment was withheld because of the interim development of brain death.

**Conclusions:**

In this patient, who presented with severe AS, the administration of catecholamine to stabilize the blood pressure probably increased the intraventricular pressures considerably despite apparently normal measurements of the central aortic pressure. IABP, temporary pacemaker, or both are recommended instead of intravenous catecholamines for patients with AMI complicated with significant AS to stabilize hemodynamic function during angioplasty.

**Electronic supplementary material:**

The online version of this article (doi:10.1186/s12872-016-0302-7) contains supplementary material, which is available to authorized users.

## Background

Left ventricular wall rupture remains a major lethal complication of acute myocardial infarction despite the recent progress in primary percutaneous coronary intervention era. The incidence of cardiac rupture has been reported to be 1.7–4.8 % of patients with acute myocardial infarction (AMI) [1–3]. Hypertension is a well-known predisposing factor of cardiac rupture after myocardial infarction [1, 4, 5]. Although a few reports have described the intraventricular hypertension associated with the increased risk of myocardial rupture in the presence of significant aortic stenosis (AS), [6–8] none were able to perform left ventriculography during percutaneous angioplasty for a patient with AMI and AS complicated by cardiac rupture because of its emergency and difficulties. We discuss this case in the context of a) the usefulness of left ventriculogram for identifying the site of left ventricular wall rupture, and b) a problem pertaining to catecholamine use for a patient presenting with acute myocardial infarction and significant aortic stenosis.

## Case presentation

**Fig. 1:**
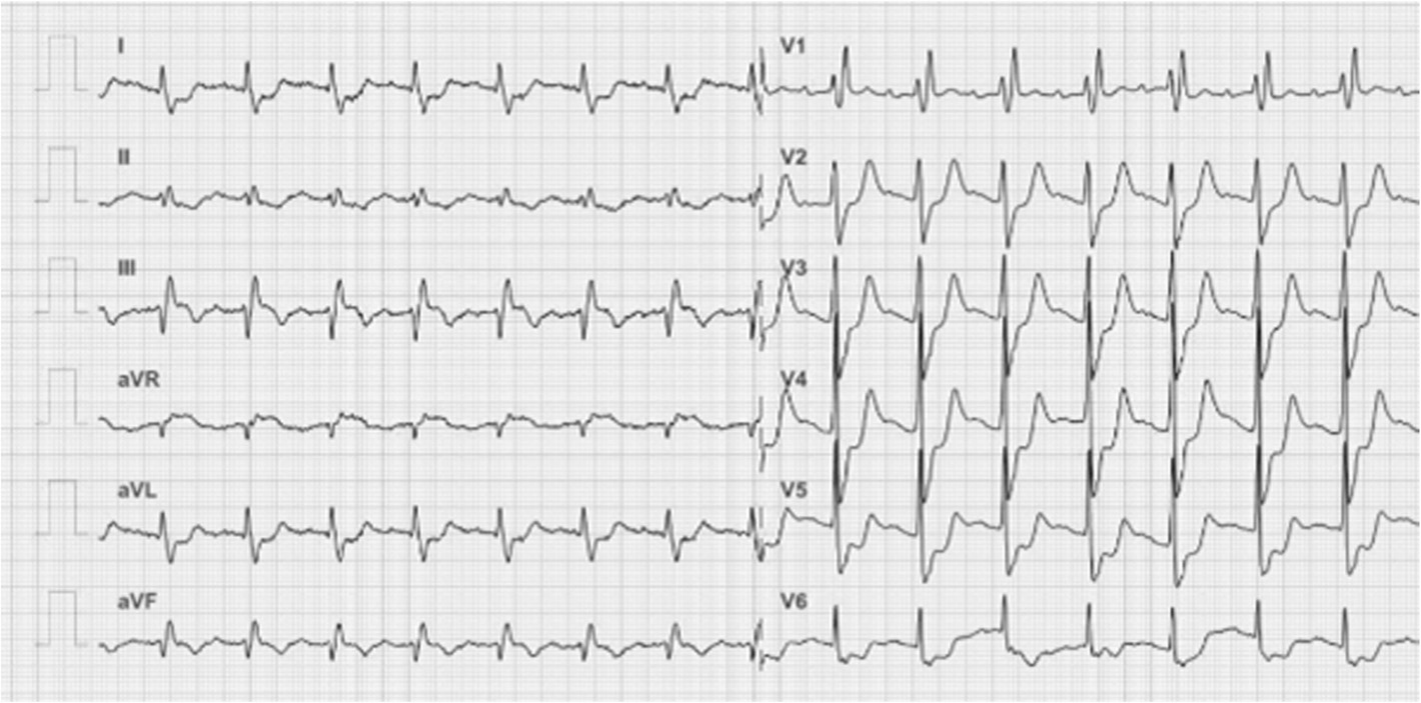
Twelve-lead electrocardiogram. Pattern of acute myocardial infarction with infero-posterior ST-segment-elevation

**Fig. 2:**
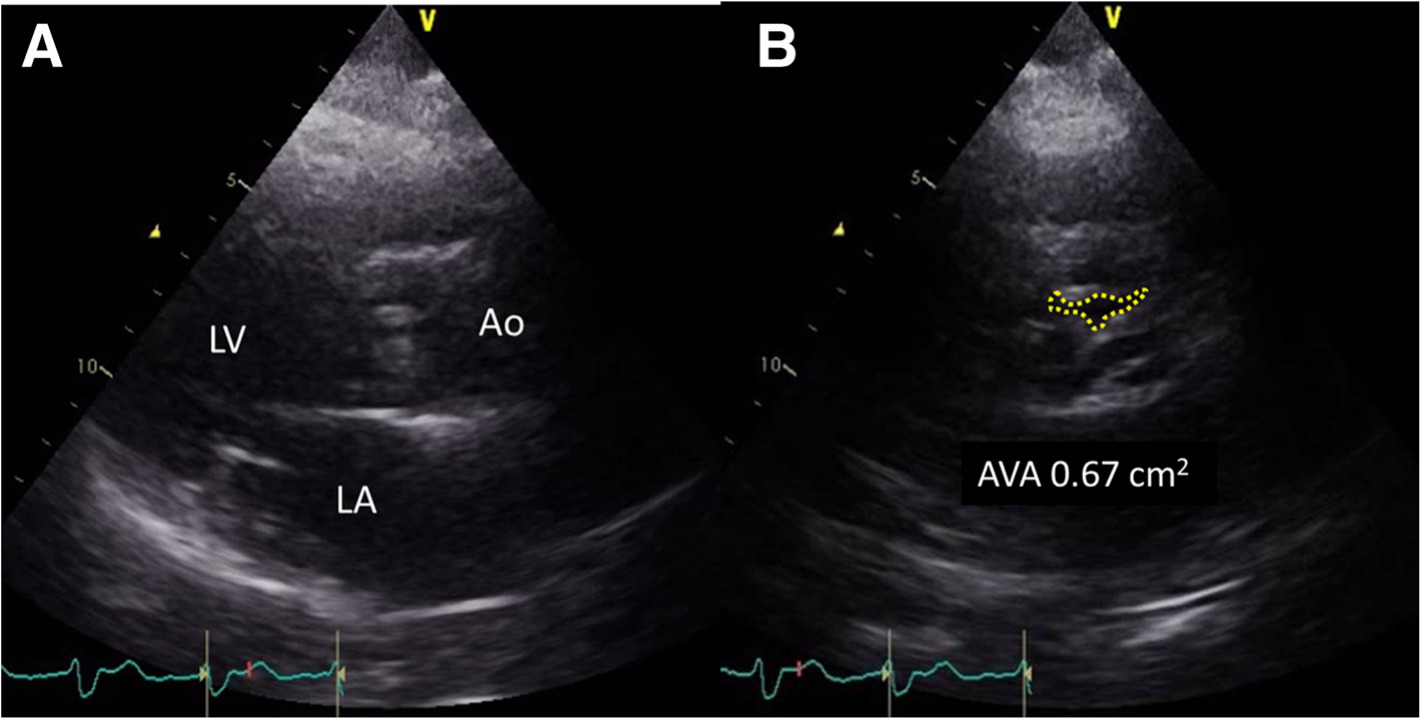
Pre-procedure transthoracic echocardiogram. **a**. Parasternal long axis view of severe aortic stenosis. **b**. Parasternal short axis view of 0.67-cm^2^ aortic valve, consistent with severe stenosis

**Fig. 3:**
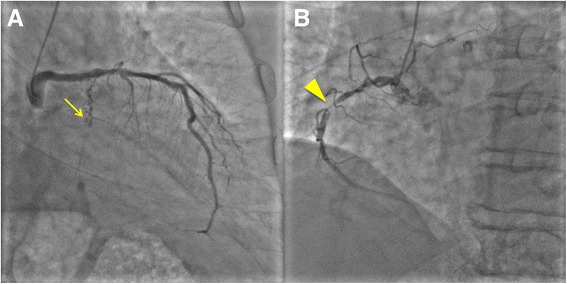
Emergency coronary angiography. **a**. Right anterior oblique caudal view of chronic occlusion of the proximal left circumflex artery, with collaterals originating from a diagonal branch. **b**. Left anterior oblique view of 99 % stenosis of the mid right coronary artery with Thrombolysis In Myocardial Infarction grade 2 flow

An 87-year-old man with histories of severe AS and diabetes mellitus was admitted to our hospital complaining of cold sweat and chest pain of 4 h duration. His heart rate was 98 bpm, blood pressure (BP) 94/57 mmHg and oxygen saturation on room air 98 %. A systolic murmur in the aortic area and a 3^rd^ heart sound were audible on auscultation. The 12-lead electrocardiogram (Fig. 1) showed 1^st^ degree atrioventricular block, complete right bundle branch block and ST segment and T wave changes consistent with an infero-posterior AMI. The white blood cell count was 13.4 × 10^3^/μl, platelet count 16.9 × 10^4^/μl, hemoglobin concentration 9.5 g/dl, troponin-T 1,614 ng/l, creatine and creatine-MB concentrations 681 and 38.9 IU/l, respectively, and the serum C-reactive protein concentration 1.30 mg/dl. A transthoracic echocardiogram showed a severely hypokinetic infero-posterior left ventricular (LV) wall, a 40 % LV ejection fraction without pericardial effusion, and a severely limited opening of the aortic valve (Fig. 2a, b). A coronary angiography performed in emergency showed a chronic occlusion of the proximal left circumflex artery and a 99 % stenosis and thrombus in the mid right coronary artery (Fig. 3a, b). During percutaneous angioplasty of the latter, hypotension and bradycardia developed at the time of balloon inflation, and etilefrine and noradrenaline were administered in order to keep optimal blood pressure and heart rate.

**Fig. 4:**
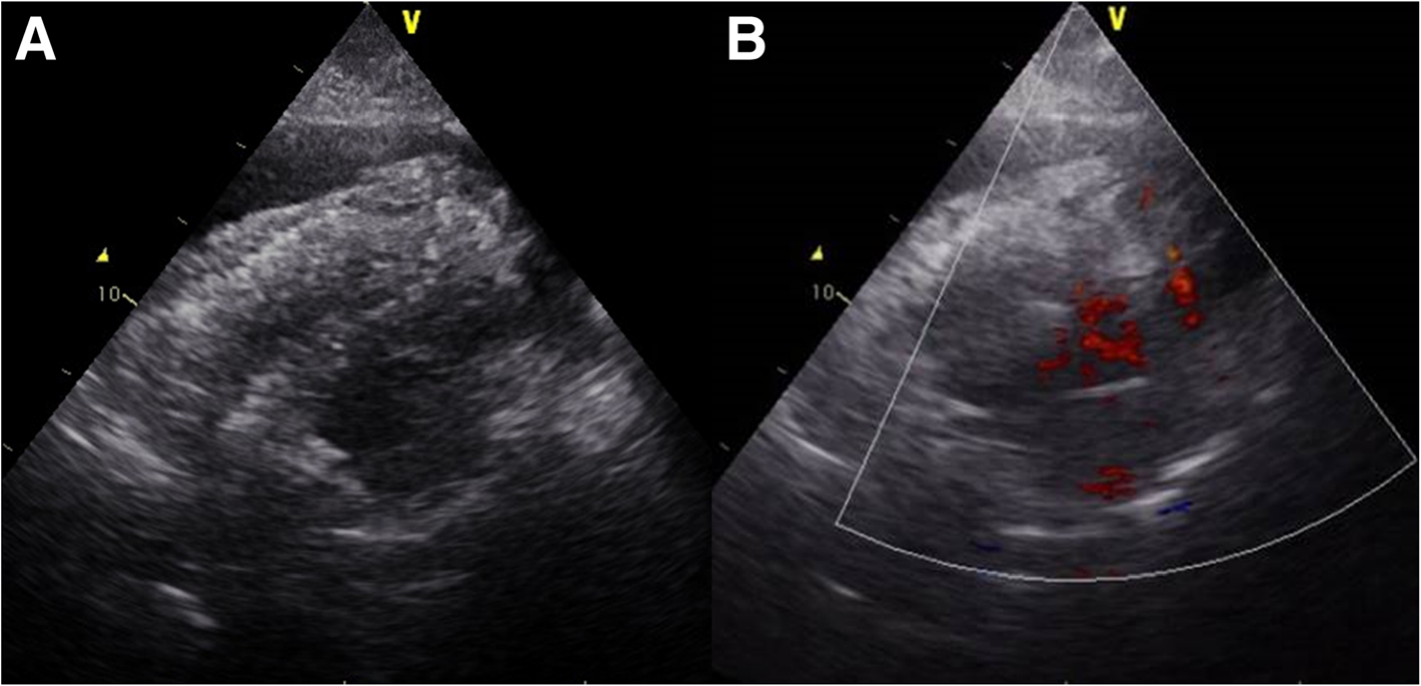
Emergent transthoracic echocardiography. **a** and **b**. Intraprocedural transthoracic echocardiography showing the presence of pericardial effusion with hematoma (arrow) and thin left ventricular infero-posterior apical wall (arrowhead)

**Fig. 5:**
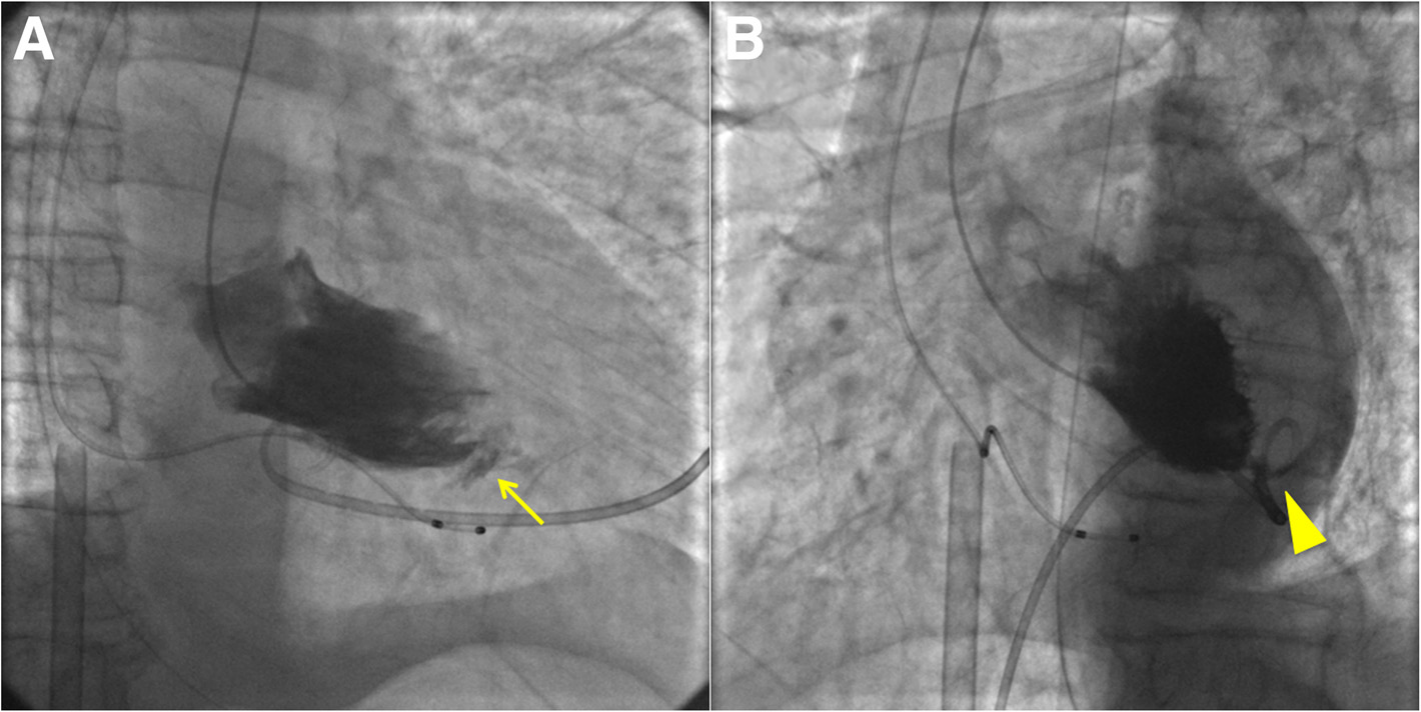
Left ventriculogram. Right (**a**) and left (**b**) anterior oblique views of the site of blowout type of infero-posterior apical wall rupture (arrow and arrowhead), with contrast spilling into the pericardial space

1 ml (once) and 2 ml (twice) intravenous boluses of a) 1 mg of etilefrine, and followed by 2 ml (4 times) of b) 1 mg of noradrenaline, each diluted in 19 ml of saline, were administered during the procedure. The aortic blood pressure rose to 120–130/60–70 mmHg during the procedure, when the patient suddenly lost consciousness and developed electro-mechanical dissociation. Cardio-pulmonary resuscitation followed by insertion of an intra-aortic balloon pump (IABP) and percutaneous cardiopulmonary support were initiated. A repeat transthoracic echocardiogram revealed the presence of a moderate pericardial effusion, though the site of free wall rupture was not distinctly visible (Fig. 4a, b). Despite an immediate pericardiocentesis followed by continuous drainage, the effusion accumulated rapidly. While waiting for an operation of cardiac surgery, we performed a left ventriculography to detect the site of the LV wall rupture, using ACIST® (ACIST Medical Systems, Eden Prairie, Minnesota, United States). Contrast agent was injected by 10 ml/s with the total amount of 20 ml. A left ventriculogram in right and left anterior oblique views clearly showed the presence of an infero-posterior apical wall rupture (Fig. 5a, b; Additional file 1: movies 1 and 2). The final coronary angiogram showed no rupture or wire perforation of the right coronary artery. The patient was transferred to the operating room. However, surgical treatment was withheld because of the interim development of brain death.

## Discussion

This is a rare case of intraprocedural blowout type of LV wall rupture diagnosed by left ventriculogram after an infero-posterior AMI in a patient with severe AS. Hypertension is one of the well-known risk factors for LV wall rupture after AMI [1, 4, 5]. In this patient, who presented with severe AS, the administration of noradrenaline and etilefrine to stabilize the BP probably increased the intraventricular pressures considerably despite apparently normal measurements of the central aortic pressure. Catecholamines increase myocardial contractility and cause peripheral vasoconstriction, both of which increase the left intraventricular pressure. On the other hand, IABP counterpulsation lowers the systolic pressure by unloading the left ventricle, and increase the mean arterial BP by diastolic augmentation [9–11]. Diastolic augmentation does not increase the left intraventricular pressure because, in absence of aortic regurgitation, the aortic valves are closed during diastole.

This case highlights the risk of intraprocedural LV wall rupture in patients presenting with AMI and severe AS by, a) increasing the intraventricular pressures, and b) attempting to preserve a normal aortic BP by the administration of catecholamines. It is also noteworthy that the use of high-speed injectors for left ventriculograms must be avoided because, by increasing the intraventricular pressure, they may rupture the left ventricle. IABP, a temporary pacemaker, or both are recommended instead of intravenous catecholamines to stabilize hemodynamic function during percutaneous coronary interventions. In this case, the insertion of an IABP and the implant of a temporary pacemaker should have been considered before proceeding with angioplasty since, in patients presenting with inferior AMI, ST segment elevation in lead aVR is not only a manifestation of basal septal infarction, but also of diffuse non-transmural apical and lateral wall ischemia from multiple vessels disease, and a sign of poor prognosis [12].

## Conclusion

Left ventriculography was useful for identifying the site of LV wall rupture after AMI complicated with AS. For such patients, IABP, a temporary pacemaker, or both are recommended instead of intravenous catecholamines to stabilize hemodynamic function during percutaneous angioplasty.

## Abbreviations

AMI, acute myocardial infarction; AS, aortic stenosis; BP, blood pressure; IABP, intra-aortic balloon pump; LV, left ventricular

## Additional file

Additional file 1: Movies 1 and 2 of left ventriculography were showed. (ZIP 7571 kb)
